# Circulating Endothelial Progenitor and Mesenchymal Stromal Cells as Biomarkers for Monitoring Disease Status and Responses to Exercise

**DOI:** 10.31083/j.rcm2312396

**Published:** 2022-12-02

**Authors:** Jared M. Gollie, Sabyasachi Sen

**Affiliations:** 1 Research & Development, VA Medical Center, Washington, DC 20422, USA; 2 Department of Health, Human Function, and Rehabilitation Sciences, The George Washington University, Washington, DC 20037, USA; 3 Department of Medicine, VA Medical Center, Washington, DC 20422, USA; 4 Department of Medicine, The George Washington University, Washington, DC 20037, USA

**Keywords:** endothelial progenitor cells, mesenchymal stromal cells, cardiorespiratory fitness, physical inactivity, resistance training, aerobic exercise, flow-mediated dilation, type 2 diabetes mellitus, prediabetes, obesity

## Abstract

Noncommunicable chronic diseases, such as obesity, cardiovascular disease (CVD), and type 2 diabetes (T2D), pose significant health challenges globally. Important advances have been made in the understanding of the pathophysiologal mechanisms and treatment of noncommunicable diseases in recent years. Lack of physical activity is a primary contributor to many noncommunicable diseases including metabolic syndrome, T2D, CVD, and obesity. Certain diabetes medications and non-pharmaceutical interventions, such as physical activity and exercise, are shown to be effective in decreasing the CVD risks associated with heart disease, stroke, obesity, prediabetes, and T2D. The ability to measure and analyze circulating adult stem cells (ASCs) has gained particular interest due to their potential to identify at-risk individuals and implications in various therapeutics. Therefore, the purpose of this narrative review is to (1) provide an overview of ASCs; specifically endothelial progenitor cells (EPCs) and mesenchymal stromal cells (MSCs), (2) describe the responses of these cells to acute and chronic exercise, and (3) highlight the potential effect of exercise on EPCs and MSCs in aging and disease. EPCs are circulating cells, abundantly available in peripheral blood, bone marrow, and umbilical cord, and are defined by cell surface markers such as CD34^+^. EPCs are expected to play an important role in angiogenesis and neovascularization and have been implicated in the treatment of CVD. MSCs are essential for maintaining tissue and organ homeostasis. MSCs are defined as multipotent heterogeneous cells that can proliferate *in vitro* as plastic-adherent cells, have fibroblast-like morphology, form colonies *in vitro*, and can differentiate into ostyeoblasts, adipocytes, chondroblasts, and myoblasts. In the presence of aging and disease, EPCs and MSCs decrease in quantity and functional capacity. Importantly, exercise facilitates EPC differentiation and production from bone marrow and also helps to promote migration and homing to the hypoxic and damaged tissue which in turn improve angiogenesis and vasculogenesis. Similarly, exercise stimulates increases in proliferation and migratory activity of MSCs. Despite the reported benefits of exercise on EPC and MSC number and function, little is known regarding the optimal exercise prescription for aging and clinical populations. Moreover, the interactions between medications and exercise on EPCs and MSCs is currently unclear. Use of ASCs as a biomarker have the potential to revolutionize the management of patients with a variety of metabolic and obesity related disorders and also pro-inflammatory diseases. Further investigation of clinical entities are urgently needed to understand the implications of interventions such as exercise, diet, and various medications on EPC and MSC quantity and function in aging and clinical populations.

## 1. Introduction

The World Health Organization (WHO) estimates nearly 450 billion people are suffering from diabetes globally [1]. As per the Centers for Disease Control and Prevention (CDC), nearly 96 million Americans have prediabetes with more than 37 million having diabetes [2]. Individuals with prediabetes are at high risk for developing type 2 diabetes (T2D), heart disease, and stroke [2]. The CDC also estimate that in the United States, 1 out of 3 adults suffer from high blood pressure, 1 out of every 20 deaths are due to stroke, and 1 out of every 4 deaths are due to coronary artery disease (CAD) [3–5]. Overweight and physical inactivity are two of the leading preventable risk factors for T2D. Being overweight or obese increases the risk for T2D, with 89.8% being overweight or having obesity (i.e., body mass index of 25 kg/m^2^ or higher) [2]. In addition, 34.3% of individuals diagnosed with T2D were classified as physically inactive, defined as getting less than 10 minutes a week of moderate or vigorous activity in each physical activity category of work, leisure time, and transportation [2]. The economic costs associated with T2D and obesity are substantial, being estimated at $327 billion for diagnosed T2D alone in the United States population [6]. These figures stress the importance of cardiometabolic complications associated with T2D and obesity and the potential implications on health care costs.

Lack of physical activity is a primary contributor to many noncommunicable diseases including metabolic syndrome, T2D, cardiovascular disease (CVD), and obesity [7–10]. Robust evidence supports that high amounts of sedentary behavior increase the risk for all-cause and CVD mortality and T2D [11–16]. For example, an inverse, non-linear dose-response relationship is observed between long-term leisure-time physical activity and all-cause and CVD mortality when assessed during up to 23-years of follow-up [14]. In adults, exercise capacity and energy expenditure are shown to be stronger predictors of all-cause mortality when compared to smoking, hypertension, obesity, and T2D [17]. Recently, Kokkinos *et al*. [15] showed that cardiorespiratory fitness is inversely associated with mortality in a graded fashion across age, sex, and race in a cohort of 750,302 United States Veterans during a median follow-up of 10.2 years. The lowest risk for mortality was seen at approximately 14 metabolic equivalents (METs) for men and women, with the risk for least fit individuals being 4-folder higher than the extremely fit individuals [15]. In addition to cardiorespiratory fitness, sufficient levels of muscular fitness is also found to have protective effects on all-cause and cancer mortality in healthy middle-aged men, men with hypertension, and patients with heart failure [18]. Importantly, it has been suggested that possessing higher muscular fitness may improve, to some extent, the adverse cardiovascular profile of overweight and obese individuals [18]. Despite the known benefits of engaging in physical activity and exercise, only 1 in 4 adults are estimated to meet the recommended levels of physical activity in the United States [19]. Among 383,928 adults (aged 18–80), only 23.5% were found to meet the physical activity guidelines of combined aerobic activity and muscle-strengthening activity [20]. Those individuals less likely to meet the physical activity guidelines tend to be older, women, current smokers, and have poorer self-rated health and lower education/income [20].

Certain diabetes medications and non-pharmaceutical interventions, such as physical activity and exercise, are shown to be effective in decreasing the CVD risks associated with heart disease, stroke, obesity, prediabetes, and T2D [10,21,22]. The United States based study Diabetes Prevention Program (DPP), lifestyle intervention significantly reduced the chances of progressing from prediabetes to T2D and risk of developing CVD [23]. According to the CDC, physical activity not only reduces the risk of developing overt T2D and CVD, but also helps to reduce body weight in individuals with obesity and has even been shown to reduce the risk of developing certain cancers associated with obesity [24]. Physical activity increases life expectancy irrespective of age, ethnicity, body shape or body mass [24,25]. Compared to those who did not meet the physical activity guidelines, individuals who engaged in muscle-strengthening activities or aerobic activities were found to be at reduced risk of all cause mortality with larger survival benefits found in those engaged in both muscle-strengthening and aerobic activities [26]. Similar patterns were observed for cause specific mortality from CVD, cancer, and chronic lower respiratory tract diseases [26]. Importantly, evidence from a metaepidemiological study of 16 meta-analyses found exercise interventions to have similar benefits of many drug interventions for the secondary prevention of coronary heart disease, rehabilitation after stroke, treatment of heart failure, and prevention of T2D [27].

Currently, diabetes medications only evaluate the hypoglycemic effect of a medication and does not always evaluate its potential to improve endothelial function and regeneration. At best, only surrogates of endothelial function are used rather than evaluating cells of endothelial lineage or even hematopoietic lineage, pre- and post-medication. Historically, the U.S. Food and Drug Administration (FDA) did not require information regarding the effects of diabetes medications on cardiometabolic health although in the last 3–5 years that trend appears to be changing. Similarly, the standard practice for monitoring the effect of exercise in clinical practice is by assessing vascular structure and function using non-invasive measures (i.e., vessel size, blood pressure, flow-mediated dilation) or by analyzing plasma or serum biochemistry focusing on surrogates of endothelial function (i.e., interleukins, high-sensitivity C-reactive protein (hs-CRP), lipid profile). However, it may be more informative to directly study cells, such as adult stem cells (ASCs) of hematopoietic lineage, as these cells may serve as a valuable biomarker to detect and monitor the effect of disease status. Furthermore, the evaluation of ASCs in response to exercise may provide insight into the cellular mechanisms underlying the associated health benefits. Therefore, the purpose of this narrative review is to (1) provide an overview of ASCs; specifically endothelial progenitor cells (EPCs) and mesenchymal stromal cells (MSCs), (2) describe the responses of these cells to acute and chronic exercise, and (3) highlight the potential effects of exercise on EPCs and MSCs in aging and disease populations.

## 2. Adults Stem Cells

Stem cells are undifferentiated cells which can differentiate into specialized or specific cell type, tissue or organ. Predominantly, natural or non-genetically modified stem cells are divided into embryonic and somatic or ASCs. ASCs hold promise both as a regenerative tool, in a proapoptotic (and pro-inflammatory) state of prediabetes and T2D but also as a biomarker to monitor progression of a disease process from prediabetes to T2D, at a cellular level. For the purposes of this review, we will concentrate primarily on two types of ASCs, one is hematopoietic cells that may be precursor to mature EPCs and MSCs. Peripheral blood contains approximately 1% of mononuclear cells as EPCs. EPCs are circulating cells, abundantly available in peripheral blood, bone marrow, and umbilical cord. EPCs have been defined by cell surface markers such as CD34^+^ (a cell surface marker to delineate a cell that has progenitor-like capabilities). The cells that have dual marks such as CD34^+^ plus kinase domain receptor (KDR), or vascular endothelial growth factor-receptor-2 (VEGFR-2) have more markers that indicate a progenitor cell that has vascular cell-like properties. Other progenitor markers include CD133 and c-kit positivity. These cells are expected to have high mRNA gene expression for typical endothelial cells such as endothelial nitric oxide synthase (eNOS) and endothelial cell specific clotting factor agents such von-Willebrand’s factor (vWF). Therefore, EPCs that have mature endothelial cell-like properties of VEGFR-2, eNOS, and vWF positivity, are expected to play an important role in angiogenesis and neovascularization and have been implicated in the treatment of CVD [28–30].

MSCs are essential for maintaining tissue and organ homeostasis. MSCs are defined as multipotent heterogeneous cells that can proliferate *in vitro* as plastic-adherent cells, have fibroblast-like morphology, form colonies *in vitro*, and can differentiate into ostyeoblasts, adipocytes, chondroblasts, and myoblasts [31–36]. Sources of obtaining MSC can vary from umbilical cord blood, bone marrow, adipose tissue, pancreatic islet, fetal liver and even the lung tissue [37,38]. MSCs express CD105, CD73 and CD90 but not CD45, CD34, CD14, or CD11b, CD79a, or CD19 and HLA-DR surface molecules [36]. This is because the latter cell surface markers are thought to be of endothelial cell lineage. The mobilization and homing of MSCs (i.e., the migration and arrest within the vasculature of a tissue followed by transmigration across the endothelium and engraftment in the target tissue) is affected by systemic and inflammatory state [39]. Bone marrow and adipose tissue derived MSCs have been well established for MSC production for therapeutic purposes [31,32,34,40]. For example, MSCs have been used in clinical trials for the treatment of various CVD’s such as ischemic heart disease, cerebrovascular stroke, chronic kidney disease, and peripheral vascular disease (PAD) [34,41]. MSCs are also involved in the processes that support skeletal muscle repair in response to resistance exercise [29,42,43].

## 3. EPCs and MSCs as Biomarkers of Aging and Disease

EPCs can act as a cellular biomarker that is more reliable than commonly used clinical serum-based markers for estimating and following endothelial dysfunction in aging, early T2D and prediabetes subjects, pre- and post-exercise, and even in response to medications [44–51]. Older adults are shown to have lower baseline levels of HSC, EPC, angiogenic T cells, as well as chemokine receptor 4 expressing circulating angiogenic cells (CXCR4-expressing CACs) [52–55]. Similarly, baseline counts of hematopoietic stem cells (HSCs), EPCs, and expression of CXCR4 and CXCR7 were significantly lower at rest in T1D group compared to healthy control group [56,57]. Older adults demonstrated endothelium-dependent dilation of the brachial artery when compared to young healthy individuals. While there was no differences in the number of circulating EPCs; lower survival, migration, and proliferation was observed suggesting functional impairment of EPCs in older adults [58].

**Fig. 1.**
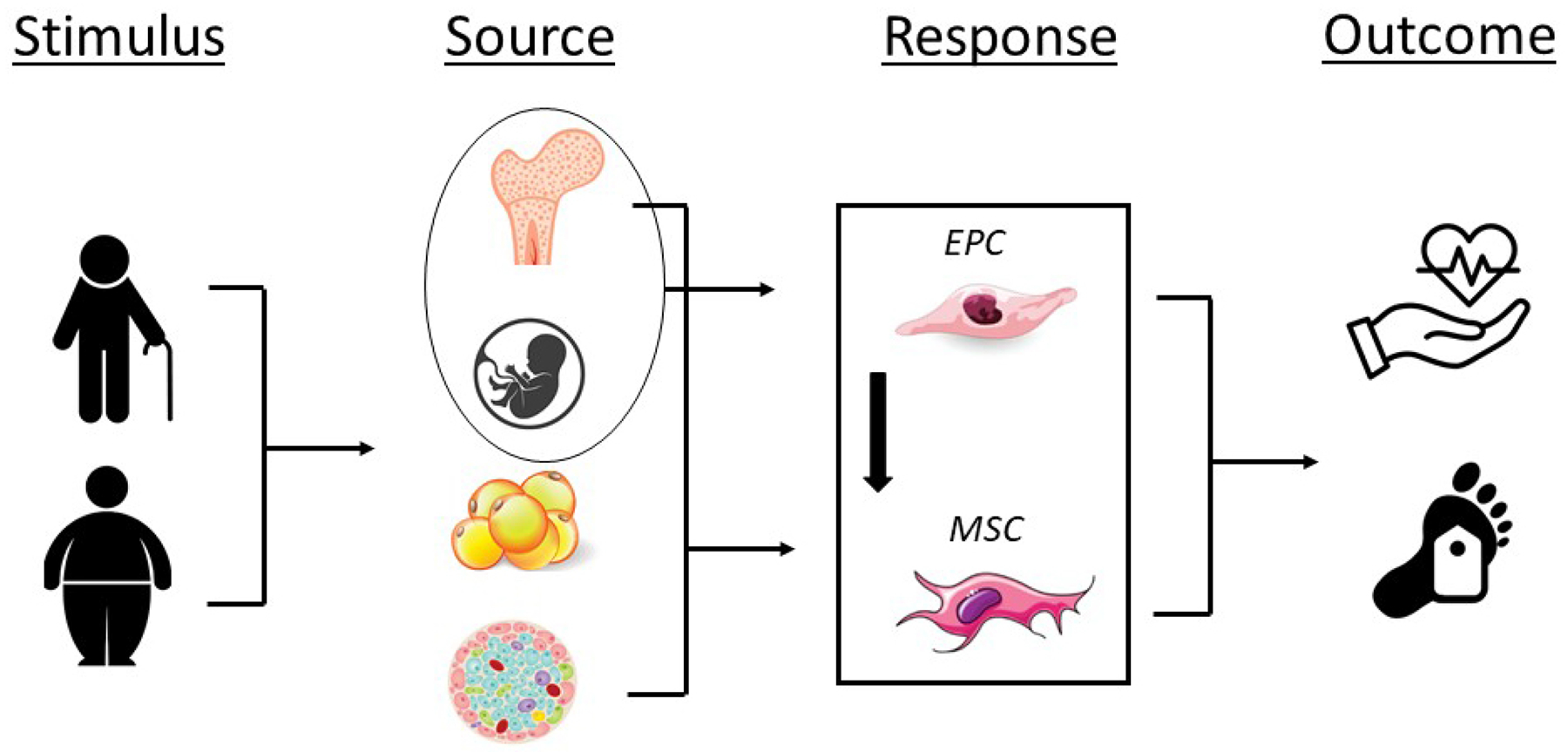
Impact of aging, obesity, and disease on endothelial progenitor (EPCs) and mesenchymal stromal cell number (MSCs) and/or function and the potential for adverse cardiovascular events and all-cause mortality. Stimulus; aging, obesity; Source, bone marrow, umbilical cord blood, adipose tissue, pancreatic islet; Response, decrease in EPC and MSC number and/or function; Outcome, adverse cardiovascular events, all-cause mortality.

The level of circulating CD34^+^/KDR^+^ EPCs predicts the occurrence of cardiovascular events and death from cardiovascular causes and may help identify patients at increased cardiovascular risk [59]. Using 1751 individuals from the Framingham Offspring cohort there was an inverse association between CD34^+^ circulating progenitor cells (CPCs) and all-cause mortality when adjusting for standard risk factors. CD34^+^CD133^+^ CPCs were inversely associated with cardiovascular mortality [60]. Associations of CD34^+^ and CD34^+^CD133^+^ with mortality were strongest in participants with pre-existing CVD. Similarly, CD34^+^ and CD34^+^/KDR^+^ are observed to be significantly reduced in individuals with T2D while CD34^+^ cells only were also reduced in prediabetic individuals [61]. Post-challenge glucose was found to be an independent determinant of the levels of both CD34^+^ and CD34^+^/KDR^+^ in individuals with T2D and pre-diabetes [61]. Number of circulating EPCs and the combined Framingham risk factor score were found to be strongly correlated when assessed in healthy men [62]. In addition, flow-mediated brachial-artery reactivity was significantly related to endothelial function and number of progenitor cells [62]. Importantly, the level of circulating EPCs were a better predictor of vascular reactivity than was the presence or absence of conventional risk factors in healthy men [62]. EPC counts have been found to differ between stroke patients and control subjects, with EPC count being lower in stroke patients, independent of age [63]. The level of EPCs in stroke patients was also significantly correlated with the Framingham coronary score [63]. End stage kidney disease (ESKD) patients showed markedly decreased numbers of EPCs and colonies when compared with controls [64,65]. ESKD had a decrease in EPC migratory function in response to VEGF and decrease in EPC incorporation into human umbilical vein endothelial cells. Framingham’s risk factor score of both ESKD and control group significantly correlated with the number of EPCs. Even in patients with mild stages of chronic kidney disease, EPC-mediated endogenous vascular regeneration has been shown to be impaired [66]. Thus, it appears both aging and chronic disease attenuate stem cell quantity and function, and are predictive of future adverse outcomes (Fig. 1).

## 4. Current Physical Activity and Exercise Recommendations for Adults

Physical activity and exercise are essential for the maintenance and improvement of health and function [24–26]. Physical activity is defined as any bodily movement produced by skeletal muscles that results in an increase in caloric requirements above resting energy expenditure [67]. Exercise refers to a specific type of physical activity consisting of planned, structured, and repetitive bodily movement to improve and or maintain one or more components of physical fitness [67]. Aerobic activities, which consist of repetitive rhythmic movements such as walking, running, cycling, and swimming, are most often prescribed for targeting cardiovascular health. Muscle-strengthening activities (i.e., resistance exercise) include weight machines, free weights, resistance bands, and body-weight exercise and are prescribed for neuromuscular health. For older adults and adults living with chronic diseases, engaging in at least 150 minutes (2 hours and 30 minutes) to 300 minutes (5 hours) per week of moderate-intensity, or 75 minutes (1 hour and 15 minutes) to 150 minutes (2 hours and 30 minutes) a week of vigorous-intensity aerobic physical activity, or an equivalent combination of moderate- and vigorous-intensity aerobic physical activity is recommended [19,67]. In addition, adults are encouraged to combine aerobic activity with 2 or more days per week of muscle-strengthening activities of moderate or greater intensity involving all major muscle groups [19,67]. While meeting the physical activity and exercise recommendations are preferred, simply increasing the amount of physical activity and exercise above sedentary levels will confer health benefits even if the recommended guidelines are not achieved [68,69]. Despite the importance of physical activity and exercise for maintaining or improving overall health, limited data exists on the effects of physical activity and exercise on EPCs and MSCs.

## 5. Acute Effects of Exercise on EPCs and MSCs

**Fig. 2.**
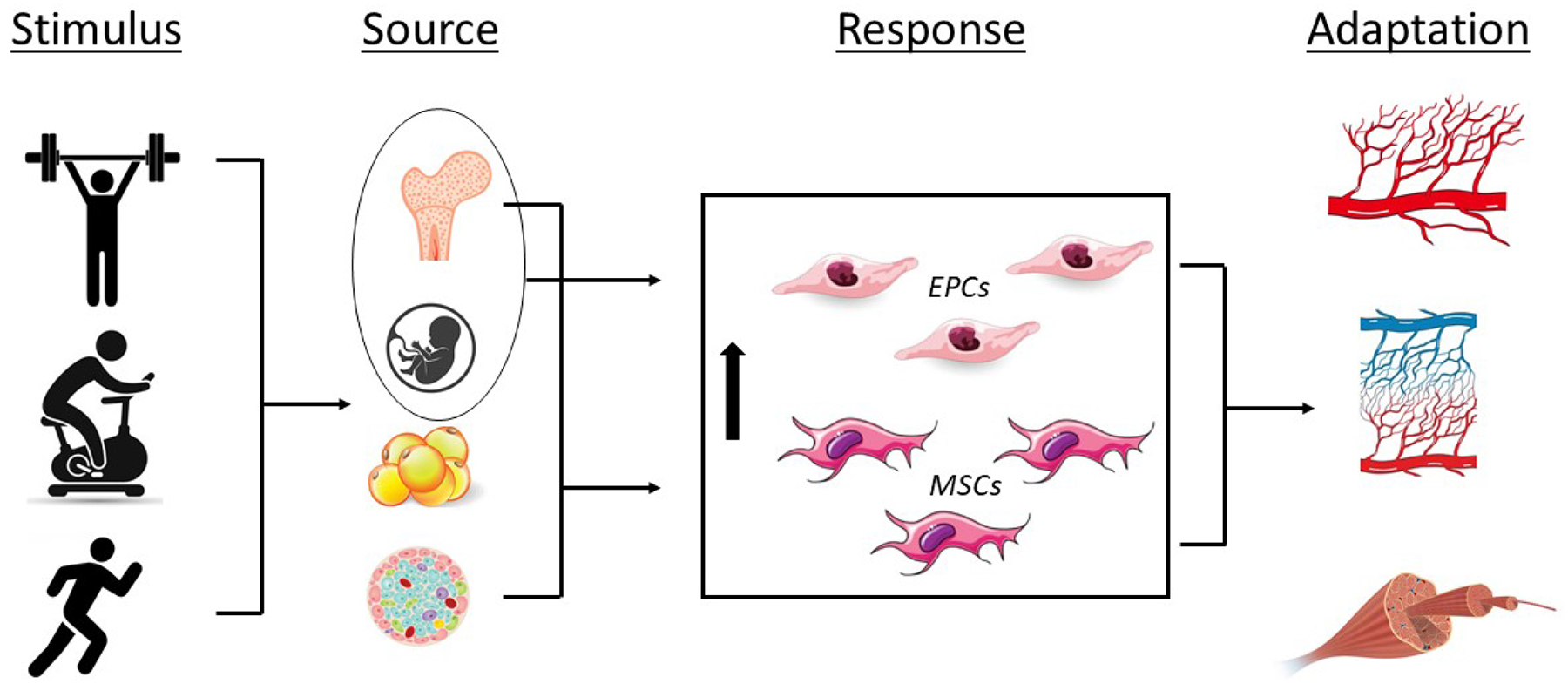
Effects of exercise on endothelial progenitor (EPCs) and mesenchymal stromal cells (MSCs) and the associated physiological adaptations. Stimulus; resistance exercise, cycling, running; Source, bone marrow, umbilical cord blood, adipose tissue, pancreatic islet; Response, increase in EPC and MSC number and/or function; Adaptation, angiogenesis, capillarization, skeletal muscle tissue repair.

Studies investigating the responses of EPCs and MSCs show transient increases in cell numbers in response to exercise (Fig. 2) [29,70–72]. Ferentinos *et al*. [70] conducted a systematic review and meta-analysis examining acute effects of different forms of exercise on circulating EPCs in healthy populations and found that prolonged endurance and resistance exercise had the most profound effect on circulating EPCs followed by maximal exercise. In another systematic review and meta-analysis examining healthy adults it was demonstrated that the extent and time-course of exercise-induced mobilization of circulating stem and progenitor cells differed between stem cell-subpopulations; i.e., EPCs, HSCs, and MSCs [71]. Early and non-specified stem cells were shown to increase significantly immediately after the initiation of exercise (i.e., 0–5 minutes) until 30-minutes post-exercise [71]. For EPC numbers, a significant increase was found until 12–48 hours after exercise and for HSC numbers at 0–5 minutes and at 3 hours after exercise [71]. No effect of exercise on MSC numbers was observed [71]. Importantly, these findings were not influenced by sex, intensity, or duration of the interventions assessed [71].

In young healthy trained men, circulating EPC and serum concentrations of vascular endothelial growth factors (VEGF-A, VEGF-C, and VEGF-D), granulocyte colony stimulating factor, soluble Tie-2, soluble fms-like tyrosine kinase-1, and matrix metalloproteinases (MMP-1, MMP-2, MMP-3, MMP-9, and MMP-10) were higher in the postexercise period following a muscular endurance resistance exercise program (three circuits of 15-repetitions of six exercises) [73]. Circulating EPC were unchanged at 10-minutes postexercise but higher at 2-hours postexercise while the concentration of most angiogenic factors and metalloproteinases were higher at 10-minutes postexercise [73]. In young healthy women, resistance exercise using intensities of 60%, 70% and 80% 1-repetition maximum performed for 3 sets of 12-repetitions increased circulating EPC and levels of VEGF, hypoxia-inducible factor 1-alpha (HIF-1*α*), and erythropoietin after exercise with the change in EPCs from baseline being greatest in the 80% 1-RM group reaching the highest levels at 6 hours post-exercise [74]. The change in EPCs from baseline to 6 hours post-exercise was correlated with the change in VEGF and HIF-1*α* [74].

## 6. Chronic Effects of Exercise on EPCs and MSCs

Moderate levels of physical activity (50–70% of maximal heart rate for 20-minutes daily for 5 days per week) increase circulating numbers of EPCs and increases EPC colony count formation which appears to have an anti-apoptotic effect in subjects with pre-diabetes [75]. Such an effect of EPC is likely to depend on the degree of inflammation, hyperglycemia, and intrinsic antioxidant enzyme presence in the EPC. Similarly, it is also reported that exercise helps to reduce apoptosis that is mediated by phosphatidylinositol 3 (PI3)-kinase pathway which is dependent on nitric oxide bioavailability [76]. Prostaglandin E1 (PGE1)-mediated upregulation of EPC is also linked to the improvement of EPC function and improved angiogenesis [77]. The improvement of EPC number may be related to, and even preceded by, an increase in plasma VEGF [44]. For example, in patients with CAD, exercise induces a short-term cellular ischemia which increases HIF which in turn increases EPC number (dependent on VEGF and HIF) [44].

Exercise-induced osteogenesis are observed for bone marrow-derived MSCs [78]. Data from our laboratoryrevealed that exercise promotes osteogenic differentiation of fat derived MSCs in prediabeteic Veteran’s [79]. Interestingly, bone differentiation markers including RUNX genes, alkaline phosphatase (ALPL), and osteocalcin were significantly upregulated indicating osteogenic differentiation [79]. Cook *et al*. [80] discussed how signaling pathways manipulate MSC differentiation. Both bone morphogenic protein (BMP) and WNT signaling pathways play an important role in MSC differentiation towards bone formation following exercise. WNT signaling promotes osteogenic differentiation by upregulating RUNX (an important gene associated with bone formation) and by inhibiting peroxisome proliferation-activated receptor gamma (PPARᵞ) (a gene that promotes adipogenesis). On the other hand, BMPs activate osteogenic differentiation by activating a prominent bone forming transcription factor, RUNX2 [80]. Maredziak *et al*. [81] also showed that four-week old male C57B1/6 mice, 5-weeks of treadmill exercise increases bone marrow derived MSC number. Markers associated with osteogenesis (i.e., ALPL activity, osteopontin, and osteocalcin) were also found to be increased postexercise [81].

Collectively, exercise facilitates EPC differentiation and production from bone marrow and also helps to promote migration and homing-in of the progenitor cells to the hypoxic and damaged tissue which in turn improve angiogenesis and vasculogenesis [82]. As might be expected, the observed responses of increased count following exercise wane over time. Duration of exercise undertaken on a daily basis influences circulating EPCs numbers [44]. It has been reported that intensive and moderate exercise activity for 30-minutes increases circulating EPC number. However, this outcome is not seen when the time of exercise is reduced to 10 minutes [45]. It has also been demonstrated that a maximal bout of exercise stimulates a significant shift in CD34^+^ cells toward CD34^+^/KDR^+^ cells, indicating a shift of undifferentiated CD34^+^ progenitor cell moving towards a differentiated cell with endothelium-like characteristics.

## 7. Implications of Exercise on EPCs and MSCs in Aging and Chronic Disease

In older adults and clinical populations, physical activity and exercise elicit beneficials effects on endothelial function which may be explained, in part, by responses of EPCs and MSCs [83,84]. Physical activity was associated with CD34^+^ CPCs only in individuals with CVD, a relationship that was maintained after adjustment for confounding variables [62]. Acute exercise promotes increases in stem cell numbers in older adults, however, the magnitude of response appears to be attenuated [53,54]. In chronic heart failure patients (CHF), EPC mobilization was acutely increased after high intensity interval training or moderate intensity continuous exercise training, while findings were inconclusive after maximal exercise testing performed on a cycle ergometer [85]. In CHF patients, CD34^+^/KDR^+^ EPC numbers increased within 10-minutes following graded-exercise testing and remained elevated for up to 2 hours post-exercise [86]. The initial increase was small in the CHF patients and normalized within 30 minutes. However, the evolution of CD34^+^/KDR^+^ EPC numbers over time following graded-exercise testing overall was attenuated in CHF when compared to healthy controls. Exercise influenced SDF-1alpha levels over time without relation to changes in CD34^+^/KDR^+^ EPC. Maximal exercise tests acutely increased EPCs in ischemic or revascularized CAD [85]. In PAD patients, EPC levels increased up to 24 hours post-exercise while EPC mobilization was blunted after a single exercise session in patients with compromised metabolic health [85].

Intravascular ultrasound-based studies have shown atherosclerotic plaque reduction or retraction in response to exercise [87]. It has been reported that supervised exercise sessions boost the circulating EPC counts while increasing angiogenesis and improved endothelial function thereby decreasing the incidence of atherosclerosis [88–90]. In patients with hypertension, exercise reduces dysfunction of EPC, which promotes neovascularization and improves hypertension [91]. Mechanical stress to the tissue and vasculature is posited as a primary mechanism underlying the promotion of enhanced EPC function following exercise [82]. The mechanical force resulting from exercise directly or indirectly helps in improvement of EPC number and function [82]. Chronic moderate-intensity continuous exercise is shown to have a positive effect on circulating EPCs in older sedentary individuals which was accompanied by improvements in endothelial function and arterial stiffness [70]. In response to 8-weeks of cycling exercise at 65–85% heart rate reserve (HRR), there was no effect on baseline or exercise-induced numbers of HSCs and EPCs [54,92]. Habitual physical activity in patients with CAD is associated with higher flow-mediated dilation and EPC count [93]. However, flow-mediated dilation was only related to increased habitual physical activity levels but not EPC count [93]. Patients with type I diabetes (T1D) performed 45-minutes of incline walking at 60% maximal oxygen consumption (VO_2max_). In both groups exercise increased circulating angiogenic cells however the increases were largely attenuated in the T1D group [56]. In young men with T1D, exercise did not induce changes in EPCs whereas in controls EPCs decreased after aerobic exercise and increased after resistance exercise [57]. Blood flow increased and vascular resistance decreased after resistance exercise in both groups. Reactive hyperemia increased 10-minutes after aerobic and resistance exercise in patients with T1D and controls with no differences between groups [57]. Despite increased vascular reactivity in both groups, EPCs were only affected by exercise in the controls which may indicate a blunted endothelium regenerating capacity in those with T1D [57]. Our laboratory has shown that 6-weeks of aerobic exercise improves EPC number and function, and upregulates endothelial cell-based gene expressions of critical endothelial specific genes such as e-NOS and VEGF while reducing inflammatory markers in patients with pre-diabetes [75].

Studies have showed that, similar to observations of EPCs, exercise may facilitate MSC homing into the site of injury [94]. It was reported that exercise induce homing of MSCs to extramedullary sites [95]. Often, the effect of MSC integration with host tissue and subsequent repair, increase with exercise [96]. It has also been reported that exercise increased the efficiency of MSC transplantation in cerebral ischemia in rats by inhibiting apoptosis [97]. Another study showed stromal vascular fraction, a well-known mixed population enriched with MSCs, when combined with exercise, together, help to improve pain in patients with knee osteoarthritis (OA) thereby establishing the synergistic effect of cell therapy and exercise in healing a common joint problem such as OA [98,99]. Exercise also plays a vital role in differentiation of multipotent MSCs towards various adult tissues. MSCs can differentiate into bone, muscle, cartilage and adipose tissue depending on the need of the body’s reparative process. The differentiation is also dependent on the cellular environment.

## 8. Future Directions

The use of ASCs as a means of assessing and monitor health status is an evolving area and shows promise for the advancement of clinical care. However, several questions remain before its application can truly be appreciated. The lack of standardized designations of cells creates challenges for comparing findings across different research laboratories. The definition of EPC need to carefully delineated based on cell surface markers rather than cell type using phenotypic nomenclatures. Following appropriate cell surface-based designations, functional assays and there related reviews, can then be focused on. While increases in circulating ASCs are seen in response to both aerobic and resistance exercise, the exact dosing of such interventions has yet to be determined. Moreover, the time course of local and systemic adaptations associated with ASC response to exercise in aging and clinical populations requires further investigation. Additionally, given that most patients are prescribe one or more medications depending on their existing conditions, it will be essential to determine how ASCs respond to exercise interventions in the presence of specific medications.

## 9. Conclusions

ASCs, such as EPCs and MSCs, can act as a cellular biomarker for cardiovascular diseases, metabolic diseases, chronic rheumatological diseases, and infectious diseases. Use of ASCs as a biomarker have the potential to revolutionize the management of patients with a variety of metabolic and obesity related disorders and also pro-inflammatory diseases. Exercise offers beneficial effects on the proliferation and migratory function of EPCs and MSCs. Further investigation of clinical entities are urgently needed to understand the implications of interventions such as exercise, diet, and various medications on EPC and MSC quantity and function in aging and clinical populations.

## Abbreviations

NIDDK: National Institute of Diabetes and Digestive and Kidney Diseases
WHO: World Health Organization
CDC: Centers for Disease Control and Prevention
CAD: coronary artery disease
T2D: type 2 diabetes
CVD: cardiovascular disease
METs: metabolic equivalence
hs-CRP: high-sensativity C-reactive protein
FDA: U.S. Food and Drug Administration
ASC: adult stem cells
HSC: hematopoietic stem cells
EPC: endothelial progenitor cell
MSC: mesenchymal stromal cell
KDR^+^: CD34^+^ plus kinase domain receptor
VEGFR-2: vascular endothelial growth factor-receptor-2
e-NOS: endothelial nitric oxide synthase
vWF: von-Willebrand’s factor
CPC: circulating progenitor cell
ESKD: end-stage kidney disease
MMP: matrix metalloproteinases
HIF-1*α*: hypoxia-inducible factor 1-alpha
ALPL: alkaline phosphatase
PPARᵞ: peroxisome proliferation-activated receptor gamma
BMP: bone morphogenic protein
CXCR4-expressing CACs: chemokine receptor 4 expressing circulating angiogenic cells
CHF: chronic heart failure
HRR: heart rate reserve
VO_2max_: maximal oxygen consumption
T1D: type 1 diabetes
OA: osteoarthritis
